# Estrogen deficiency accelerates lumbar facet joints arthritis

**DOI:** 10.1038/s41598-017-01427-7

**Published:** 2017-05-03

**Authors:** Hao Chen, Hai Zhu, Kai Zhang, Kangwu Chen, Huilin Yang

**Affiliations:** 1 Department of Orthopaedics, The First Affiliated Hospital of Soochow University, No. 188 Shizi Street, Suzhou Jiangsu, 215006 P.R. China; 2 Department of Orthopaedics, Affiliated Hospital of Nantong University, Xisi Road 20, Nantong Jiangsu, 226001 P.R. China; 3 Institute of Orthopaedics, Soochow University, No. 708 Renmin Road, Suzhou Jiangsu, 215006 P.R. China

## Abstract

Dramatic increase in the prevalence of lumbar facet joint (LFJ) arthritis in women around the age of menopause indicates a protective role for estrogen in LFJ arthritis. To date, there is no evidence for this indication and the mechanism of such an effect remains poorly understood. In this study, ovariectomized (OVX) mice were used to mimic the estrogen-deficient status of post-menopausal women. Micro-CT and immunohistochemistry was employed to assess the morphological and molecular changes in ovariectomy-induced LFJ arthritis. The results show that the LFJ subchondral bone mass was significantly decreased in OVX mice, with increased cavities on the interface of the subchondral bone. Severe cartilage degradation was observed in ovariectomy-induced LFJ arthritis. Increased blood vessels and innervations were also found in degenerated LFJ, particularly in the subchondral bone area. 17β-Estradiol treatment efficiently suppressed LFJ subchondral bone turnover, markedly inhibited cartilage degradation, and increased blood vessel and nerve ending growth in degenerated LFJ in OVX mice. Our study reveals that estrogen is a key factor in regulating LFJ metabolism. Severe LFJ degeneration occurs when estrogen is absent *in vivo*. Collapsed subchondral bone may be the initiation of this process, and estrogen replacement therapy can effectively prevent degeneration of LFJ under estrogen-deficient conditions.

## Introduction

Lumbar facet joints (LFJ) are a set of synovial joints between the articular processes of two adjacent spinal levels in humans. Arthritis in LFJ accounts for 15–40% of the chronic low back pain, which is prevalent in populations worldwide^1, 2^. Many risk factors for LFJ arthritis have been studied, such as age, sex, and body weight^1^. Among these, sex has been identified as an independent risk factor contributing to LFJ degeneration. Lumbar computed tomography (CT) and X-ray plain film observation studies have shown that LFJ degeneration occurs more frequently in women^3, 4^. It has also been documented that the prevalence of LFJ arthritis increases dramatically in women but not in men around the age of 50 years, which coincides with the onset of menopause^5^. Decreased ovarian production of hormones, particularly estrogen, is the major biological change during the female menopause^6^. In addition, estrogen receptor expression observed in LFJ cartilage has been found to correlate with the severity of LFJ arthritis^7^. Taken together, the collected evidence indicates that estrogen might play a pivotal role in regulating the metabolic homeostasis of LFJ.

Given this potential role of estrogen in protecting LFJ integrity, understanding the influence of estrogen on LFJ would shed light on the possible mechanisms of persistent low back pain in post-menopausal women, which in turn could be helpful in the development of therapeutic targets. In this study, we explored the association between estrogen and LFJ arthritis with investigations of the function of estrogen in LFJ integrity in an OVX mouse model.

## Results

### Estrogen deficiency leads to LFJ subchondral bone collapse

**Figure 1:**
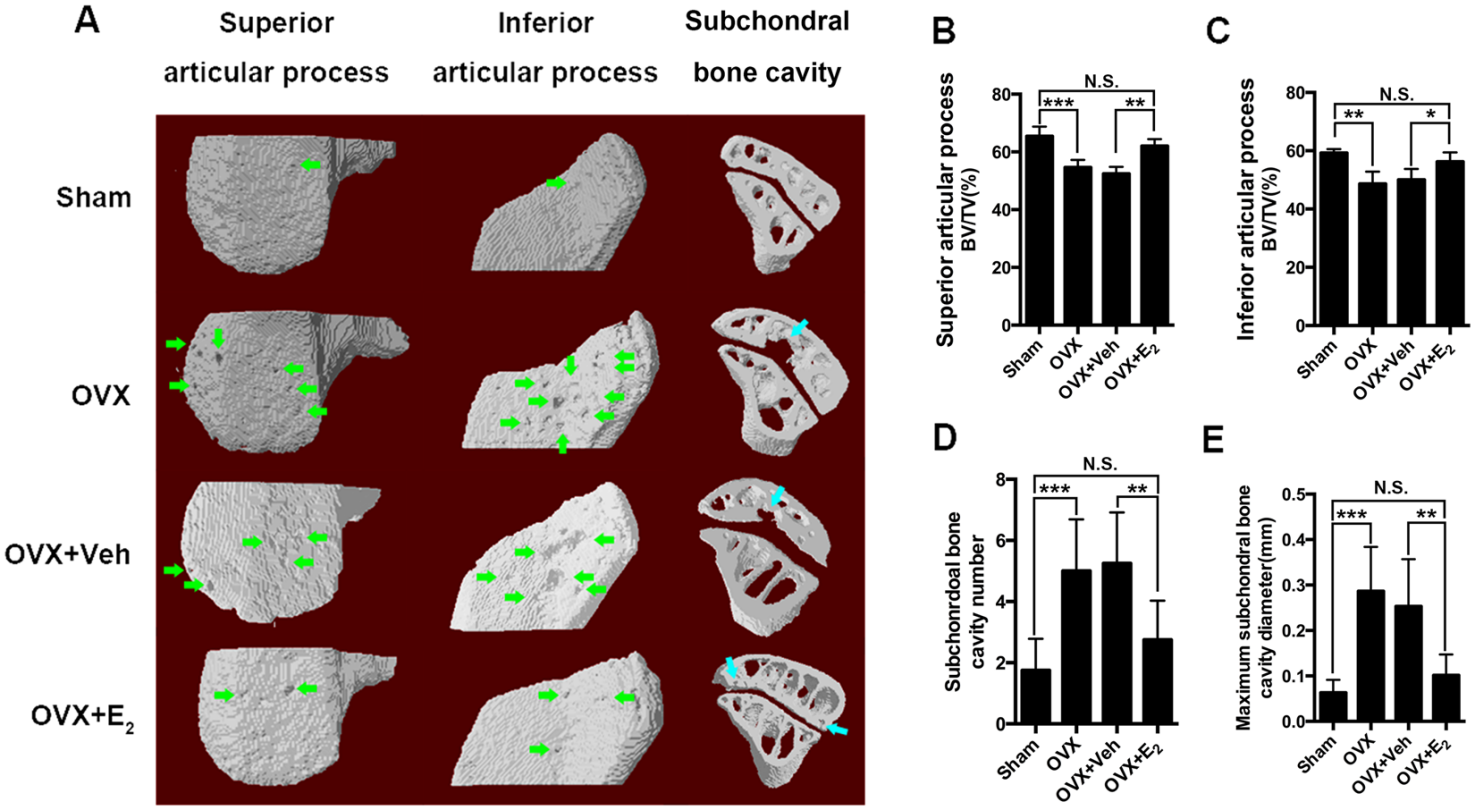
Estrogen deficiency leads to LFJ subchondral bone collapse. (**A**) Representative 3-dimensional and 2-dimensional micro-CT images of the LFJ subchondral bone in different groups. Abundant subchondral bone cavities (green and blue arrows) are shown in OVX and OVX+ vehicle (Veh)-treated groups. (**B**) Superior bone volume/tissue volume (BV/TV) of LFJ subchondral bone. (**C**) Inferior BV/TV of LFJ subchondral bone. (**D**) LFJ subchondral bone cavity numbers in different groups. (**E**) LFJ subchondral bone maximum diameters in different groups. Key: *P < 0.05, **P < 0.01, ***P < 0.001, (N.S.) not significant.

Estrogen deficiency-induced morphological changes in the subchondral bone of LFJ were shown by micro-CT scanning the lumbar spine of OVX mice (Fig. 1). Estrogen deficiency decreased the subchondral bone BV/TV ratio in both superior (OVX 54.64% vs. sham 65.41%) and inferior (OVX 48.65% vs. sham 59.22%) articular processes. As shown in Fig. 1A,B and C, 17β-estradiol (E_2_) treatment significantly prevented subchondral bone loss (superior 62.06% and inferior 56.25%) compared to the vehicle-treated OVX mice (superior 52.45% and inferior 50.03%). Intriguingly, the appearance of localized perforations on the osteochondral interface could be distinctly observed in the OVX group, whereas the interface in the sham group was integrated, with limited visible tiny perforations (Fig. 1A). The average number of instances of subchondral bone cavities in the OVX group was 5, compared to 1.5 in the sham group (Fig. 1D). The maximum subchondral bone cavity diameter in OVX mice was 0.29 mm, which was approximately 4.5-fold larger than that in the sham-operated mice (Fig. 1E). Injections of E_2_ successfully prevented estrogen deficiency-induced subchondral bone cavities formation, both in number (2.13) and maximum cavity diameter (0.10 mm) (Fig. 1D and E). These results confirm the occurrence of accelerated subchondral bone remodeling under estrogen-deficient conditions. Coincidentally, collapsed osteochondral interfaces might also have contributed to LFJ arthritis when estrogen was lost.

**Figure 2:**
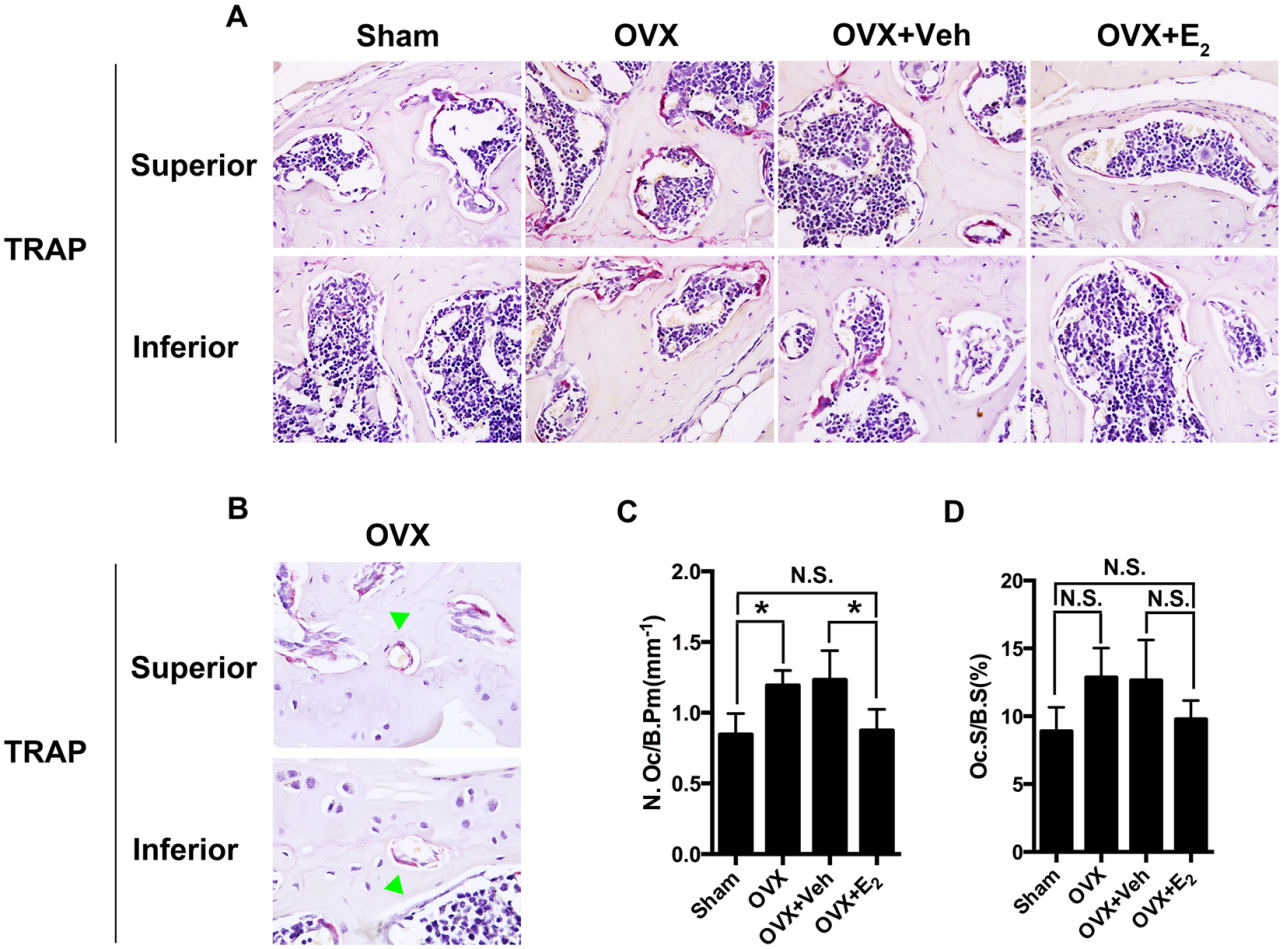
Estrogen deficiency accelerates LFJ subchondral bone resorption. (**A**) Representative TRAP staining images show abnormal bone remodeling in subchondral bone marrow in OVX and OVX+ Veh-treated groups (magnification 600×). (**B**) Increased osteoclasts are shown in subchondral bone cavities (green arrow head; magnification 1000×). (**C** and **D**) Quantitative evaluation of osteoclasts in LFJ subchondral bone. Key: *P < 0.05, (N.S.) not significant.

Bone turnover is likely to participate in the pathogenesis of joint degeneration^8^. Furthermore, micro-CT data showed collapsed subchondral bone in LFJ. We therefore explored the pathological changes with TRAP staining to visualize the osteoclasts in LFJ subchondral bone (Fig. 2). TRAP staining revealed an increased number of osteoclasts in the OVX mice (N.Oc/B.Pm 1.19 mm^−1^, Oc.S/BS 12.87%) compared to the sham-operated mice (N.Oc/B.Pm 0.85 mm^−1^, Oc.S/BS 8.89%). In addition, E_2_ treatment efficiently inhibited osteoclasts (N.Oc/B.Pm 0.90 mm^−1^, Oc.S/BS 9.78%) (Fig. 2A,C and D). Notably, increased osteoclasts were observed in the cavities on the osteochondral interface, giving a possible illustration of the formation of the cavities (Fig. 2B). Together with the micro-CT data, the ovariectomy-induced osteoclast increase may account for bone loss in the LFJ subchondral bone. This bone loss could also be associated with the collapsed osteochondral interface during the degeneration process.

### Estrogen deficiency induces LFJ cartilage degradation

**Figure 3:**
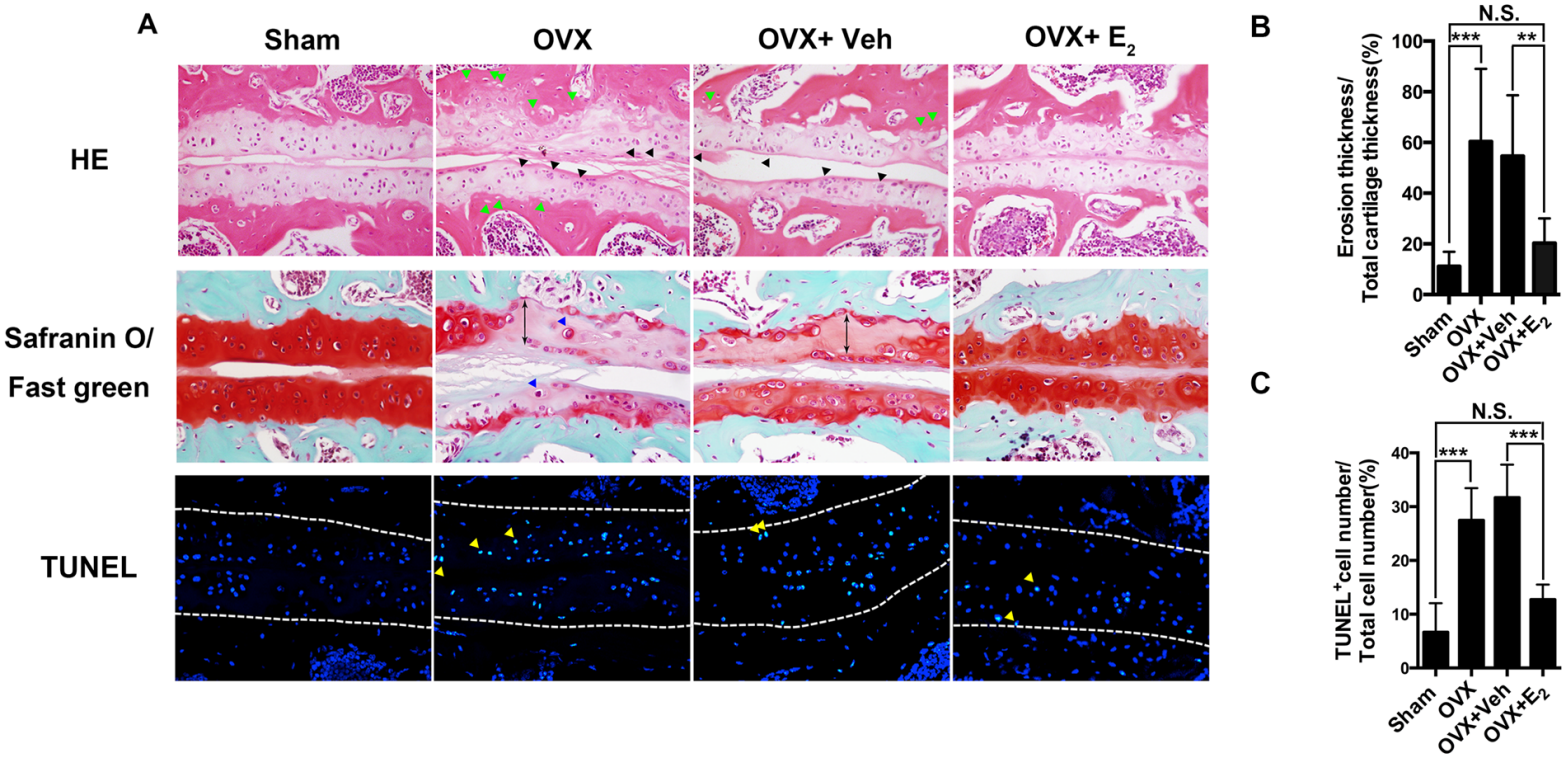
Estrogen deficiency induces severe LFJ cartilage degradation. (**A**) Staining LFJ with HE, safranin O/Fast green and terminal-deoxynucleoitidyl transferase mediated nick end labeling (TUNEL) in different groups. Chondrocyte clusters (black arrow head), hypertrophic chondrocytes (blue arrow head), osteocyte lacunaes (green arrow head) and apoptotic chondrocytes(yellow arrow head) are seen in OVX and OVX+ Veh-treated groups (magnification 400×). (**B**) Quantitative evaluation of LFJ cartilage degeneration in different groups. Cartilage erosion is labeled with double-headed arrows (magnification 600×). (**C**) Quantitative evaluation of LFJ chondrocytes apoptosis in different groups (magnification 600×). Key: **P < 0.01, ***P < 0.001, (N.S.) not significant.

Cartilage is one of the major elements in joint structure, and pathological change is always the main concern in joint degeneration. To validate the effect of estrogen on facet joints, the facet joint sections were stained with ERα. It showed that ERα was expressed in different component of LFJ, including the chondrocytes and subchondral bone marrow cells (Supplemental Fig. 1), and there was no significant difference between the expression of ERα in sham and OVX mice. As shown in Fig. 3A, the cellularity and structural abnormalities of the degenerated LFJ are clearly presented in OVX mice. Staining with HE revealed reduced cartilage layer thickness, with loss of chondrocytes, formation of multicellular cell clusters, and a less ordered arrangement of chondrocytes in the cartilage. The focal superficial matrix was condensed, and superficial fibrillation appeared. The collapsed subchondral bone with cavities and increased osteocyte death seen upon HE staining is consistent with the micro-CT data. Part of the interface between subchondral bone and cartilage in the degenerated LFJ was undistinguishable. Safrinin O/fast green staining showed that the normal architecture of the cartilage matrix was almost lost, and numerous hypertrophic chondrocytes appeared in the degenerated cartilage. Strikingly, remarkable improvement was seen in estrogen deficiency-induced LFJ arthritis following treatment with E_2_. Although mild degenerative signs could still be observed, most of the matrix in the cartilage and the cells were prevented from degeneration. To quantify the severity of cartilage degeneration, we evaluated cartilage erosion percentage (erosion thickness/total cartilage thickness). There was a dramatic increase in erosion percentage in the OVX group (60.38%) compared to the sham group (11.13%). Estrogen administration markedly lowered erosion percentage in the OVX group (20.25%), but it was still significantly higher than that in the sham group (Fig. 3B). As cell death is another possible risk factor in the pathological process of arthritis, we explored the apoptotic rate of chondrocytes in LFJ in different groups with TUNEL staining (Fig. 3A). The results showed that apoptotic rate in the sham group was only 6.65%, whereas, it increased to 27.44% in OVX group, and E_2_ treatment decreased the apoptotic rate to 12.73% (Fig. 3C). Histological analysis and TUNEL staining revealed the severe LFJ cartilage damage caused by estrogen deficiency, and indicated that E_2_ administration could effectively slow down the degeneration process.

**Figure 4:**
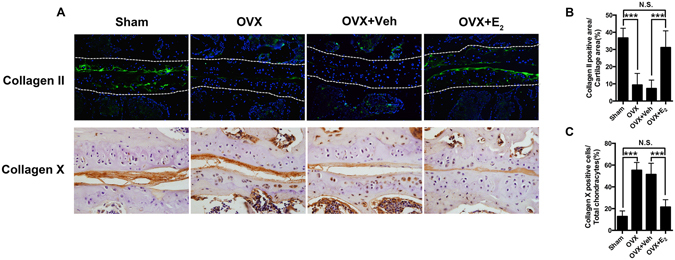
Change of type II and type X collagen expression in ovariectomy-induced LFJ cartilage degeneration. (**A**) Immunofluorescent and immunohistochemistry staining of type II and type X collagen in LFJ cartilage (magnification 600×). (**B** and **C**) Quantitative evaluation of type II collagen-positive areas and type X collagen-positive chondrocytes in LFJ cartilage. Key: ***P < 0.01, (N.S.) not significant.

Molecular regulation plays pivotal roles during the joint degeneration process. Type II and type X collagen have been widely accepted as molecular markers for cartilage evaluation^9^. In contrast to the sham group, type II collagen could hardly be observed in the LFJ cartilage in OVX mice. Nonetheless, type II collagen re-appeared after estrogen administration in OVX mice. On the contrary, type X collagen-positive cells in the cartilage markedly increased once the estrogen was lost, and E_2_ treatment effectively inhibited its up-regulation (Fig. 4). The results indicate molecular disregulation in the LFJ cartilage when estrogen was absent *in vivo*, further validating the occurrence of cartilage degradation during this process.

### Estrogen deficiency promotes blood vessel formation and innervation in LFJ

**Figure 5:**
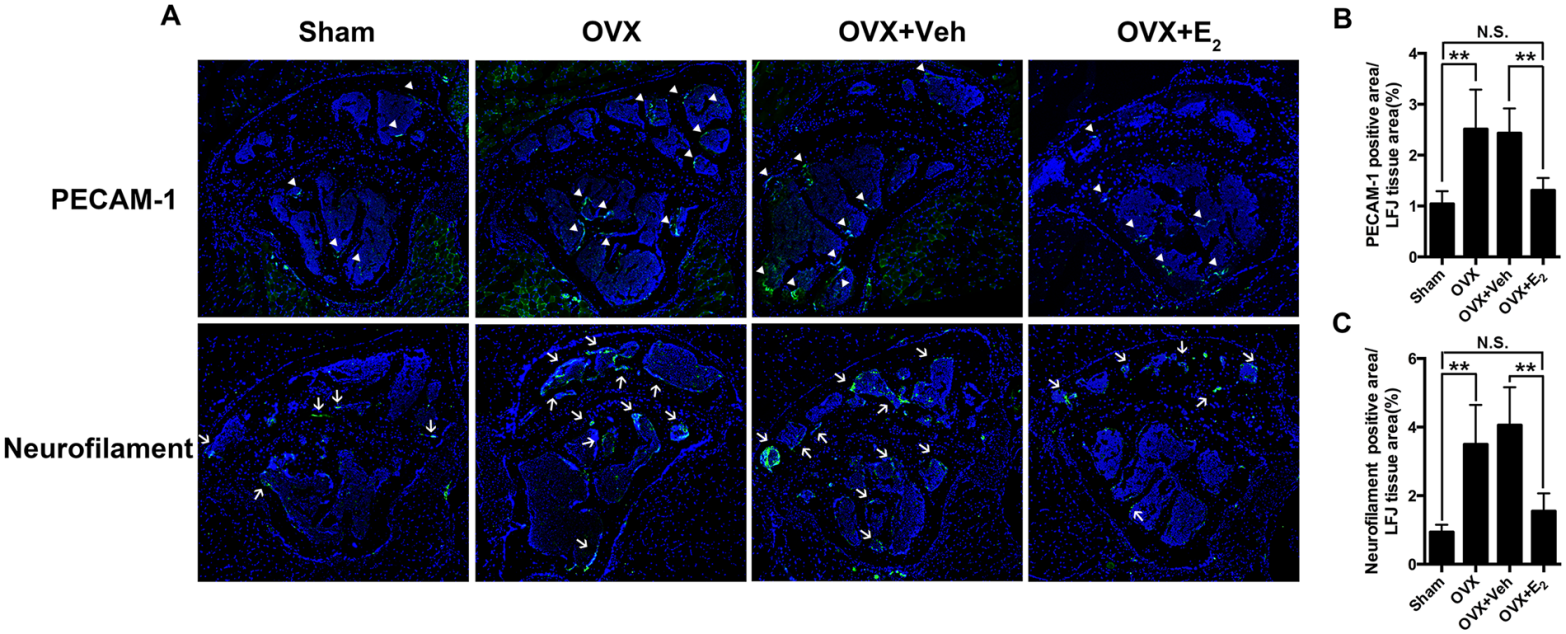
Estrogen deficiency promotes blood vessels and nerve endings innervation into LFJ. (**A**) PECAM-1-positive blood vessels (white arrow head) and neurofilament-positive nerve endings (white arrow) in LFJs from different groups. (**B** and **C**) Quantitative evaluation of PECAM-1-positive areas and neurofilament-positive areas in LFJ (magnification 600×). Key: **P < 0.05, (N.S.) not significant.

Previous studies in human facet joint samples have demonstrated that nerve endings innervate the capsule, synovial folds, and subchondral bone of facet joints^10–12^. Angiogenesis and innervation are always closely integrated, so we performed immunofluorescent staining for PECAM-1 and neurofilament in LFJ (Fig. 5). Compared with the sham group, PECAM-1-positive vessels and neurofilament-positive nerve endings increased significantly in OVX mice, whereas estrogen administration apparently diminished their growth. We noticed that the blood vessels and nerve endings were mostly innervated along the trabecular surface of the facet joints, which is in accordance with previous reports. However, only small amounts were observed in the joint capsule, which might be due to differences in the tissue areas and orientations of the sections we chose for staining. To further elucidate estrogen deficiency induced pain from facet joints, we performed grip force test. The results showed that OVX mice displayed significant impairment in the grip force test than the sham group mice, indicating the axial discomfort. After E_2_ treatment of OVX mice, the discomfort of axial low back was presented by demonstrating with enhanced grip force (Supplemental Fig. 2).

## Discussion

In this current study, we firstly analyzed the estrogen deficiency induced LFJ degeneration. Our results indicate that estrogen itself is indispensible in maintaining the normal architecture of LFJ, as severe degeneration occurred in estrogen deficient condition, and exogenous estrogen administration suppressed this pathological process and restored the integrity of LFJ to a great extent.

Subchondral bone loss was a major pathological change that we observed in ovariectomy-induced LFJ arthritis. Based on Mankin score evaluation, no difference was found in knee joint arthritis between rabbit osteoporosis models induced by ovariectomy and those induced by ovariectomy combined with methylprednisolone injection^13^, implying a limited role of osteoporosis in the joint degeneration process. In addition, increased cavity formation on the interface between articular cartilage and subchondral bone (osteochondral interface) in ovariectomy-induced LFJ arthritis has also been reported. Duncan *et al*.^14^ firstly found perforations on non-arthritic tibial plateaus, several of which even penetrated into the subchondral bone marrow. A similar phenomenon was also identified on the human tibial plateau^15^. Sander *et al*.^16^ explained in their study that instability-induced mechanical stress initiated the increase of osteoclast activity in the knee subchondral bone area just below the osteochondral interface. This increase in osteoclast activity led to a decrease in subchondral bone plate thickness and enlargement of perforations formed in the plate, which could result in higher fluid exudation towards the subchondral bone and cause subsequent damages. As shown in this study, we observed activated osteoclasts underlying the LFJ osteochondral interface in OVX mice, which might lead to the collapse of osteochondral interface and allow mechanical and biochemical interactions between subchondral bone and articular cartilage^17^.

Our results also show severe LFJ cartilage damage in OVX mice, and indicate that estrogen replacement therapy effectively slows down this process. Previous studies have identified estrogen receptors on most joint components, including cartilage, bone, ligaments, and synovium^18–22^, indicating a regulatory role for estrogen in these tissues. Because cartilage damage is the major radiological and pathological manifestation observed in joint degeneration, several studies have explored the influence of estrogen on cartilage metabolism. E_2_ was shown to enhance glycosaminoglycan synthesis, which is necessary for normal cartilage and joint function, in cultured rabbit chondrocytes^23^. Furthermore, E_2_ can suppress cyclooxygenase-2 expression in the chondrocytes from bovine joints, which protects the cells from reactive oxygen-induced damage^24, 25^. *In vivo* studies yielded contradictory data on the effects of estrogen on knee joint cartilage turnover. Several studies using histological evaluation and urinary marker collagen type II degradation product (CTX-II) measurement^26–28^ report that estrogen efficiently inhibits knee cartilage turnover in OVX rats. Conversely, intra-articular injection of estrogen is reported to further break down the cartilage structure in estrogen-deficient rabbits^29, 30^.

In our study, increased angiogenesis and neurogenesis were observed in OVX induced LFJ degeneration. David *et al*.^31^ revealed that osteochondral angiogenesis, which is regulated by a pro-angiogenesis micro-environment within the subchondral bone marrow, is increased in rheumatoid arthritis and osteoarthritis than non-degenerated knee joints. Fibrovascular replacement of marrow tissue is thought to be associated with new blood vessels formation in degenerated subchondral bone, in which the angiogenic cytokines including interleukin (IL)-1α, IL-8, IL-10, vascular endothelial growth factor and platelet-derived growth factor are up-regulated^32–34^. Neovascularization is always accompanied by innervation, which has been identified in vascular channels in degenerated subchondral bone with immunoreactive staining. Furthermore, nerve growth factor was also found to co-localize with increased nerve fibers in knee joint degeneration, which contributes to arthritis pain sensation by sensitizing the peripheral nerve endings.

In conclusion, our study reveals that estrogen is a key factor that regulates LFJ metabolism. Severe LFJ degeneration, both in cartilage and subchondral bone, occurs when estrogen is absent *in vivo*. Collapsed subchondral bone might enable the initiation of this process, and estrogen replacement therapy can effectively prevent LFJ from degeneration in estrogen-deficient conditions.

## Materials and Methods

### Animals

32 female C57Bl/6j mice aged 12 weeks were used in the study. The animals were randomly assigned into four groups of eight mice each. Three groups of mice underwent bilateral ovariectomy with a dorsal approach, and the fourth group underwent a sham operation. One of the OVX mice groups received subcutaneous injections of 10 μg/kg 17β-estradiol (Sigma, St. Louis, MO) starting 3 days post-ovariectomy. Another of the OVX mice groups received subcutaneous injections of vehicle. The E_2_ and vehicle injections were administered 5 days/week for 8 weeks. The design of the mice groups were shown in Supplementary Table 1. The mice were sacrificed following the standard protocol, and all the lumbar spines were harvested and fixed in 10% buffered formalin for the following micro-CT and histopathological analysis. The procedures involving animals and their care conformed to the U.S. National Institute of Health guidelines (NIH Pub. No. 85-23, revised 1996). All animal-related procedures and experiments were approved by the Ethics Committee of the First Affiliated Hospital of Soochow University.

### Micro-CT analysis of LFJ subchondral bone

The fixed lumbar spines were scanned by micro-CT with a SkyScan scanner(SkyScan, Aartselaar, Belgium). The isometric resolution was set at 6 μm, and the X-ray energy setting was 80 kV, 100 μA. The bone volume (BV)-to-tissue volume (TV) ratio was used to evaluate the subchondral bone mass of the LFJ. Facet joint arthritis distribute unevenly throughout the spine. The classic radiographic features of facet joint arthritis are most common at L4–L5, followed by L5–S1^35^. Therefore, micro-CT analysis was performed on the L4–L5 region of the LFJ. The number and maximum diameters of the subchondral bone cavities, which were defined as tunnel-like corridors on the osteochondral surface, were also evaluated^36^.

### Histological and pathological analysis

The fixed spine samples were decalcified in 10% EDTA (Sigma, St. Louis, MO) for 7 days and then embedded in paraffin. Next, 5-μm thick transaxial-oriented sections of the L4–L5 facet joints were subjected to staining with hematoxylin and eosin (HE) (Dako, Glostrup, Denmark), Safrinin O/fast green (Sigma), tartrate-resistant acid phosphatase (TRAP) (Sigma), and terminal-deoxynucleoitidyl transferase mediated nick end labeling (TUNEL) according to the manufacturer’s protocols. As previously described for immunostaining^37^, paraffin was removed from all the sections before antigen retrieval (Dako, Glostrup, Denmark) and blocking steps. The sections were then incubated overnight at 4 °C with antiERα(Santa Cruz, Dallas, TX), anti-type II collagen (Abcam, Cambridge, MA), anti-type X collagen (Abcam), anti-PECAM-1 (Santa Cruz, Dallas, TX), and anti-neurofilament (Abcam) primary antibodies. Corresponding second antibodies were added onto the sections after the sections were rinsed three times in phosphate-buffered saline (PBS). For immunohistochemistry, the sections were then counterstained with hemotoxylin (Dako). For immunofluorescence, the sections were counterstained with 4′,6-diamidino-2-phenylindole (DAPI; Sigma). All the sections were observed under the microscope (Zeiss) and scored in a blinded fashion. Cartilage erosion thickness-to-total cartilage thickness ratio was used to measure the cartilage degeneration in our OVX mice model^8^. To quantification of PECAM-1 and Neurofilament staining, ImageJ was applied. The positive staining part was drawn and defined as regions of interest. The area of positive staining versus the total area of facet joint was used to reflect the amount of vessels or nerve that innervated into the facet joint.

### Behavioral measurement of axial low back pain

Axial discomfort was measured by employing the grip force assays as previous described^38^. Briefly, the animal was gently stretched while gripping a bar with its forepaws until the point of release, and the force, in grams, was recorded. Grip force was measured 3 times in each animal and expressed as the average in grams.

### Statistical analysis

All the data were analyzed by SPSS software and were presented as mean ± standard deviation. Statistical differences were analyzed by unpaired *t* test and analysis of variance with a post-hoc test (Tukey-Kramer test). A P value of ≤0.05 was considered significant.

### Availability of materials and data

The datasets generated during and/or analysed during the current study are available from the corresponding author on reasonable request.

## Electronic supplementary material

Supplemental figures
