# Supplementary material for: Estrogen deficiency accelerates lumbar facet joints arthritis
Source: Sci Rep. 2017 May 3;7:1379. doi: 10.1038/s41598-017-01427-7 (PMC5431109; doi:10.1038/s41598-017-01427-7)

Estrogen deficiency accelerates lumbar facet joints arthritis

Hao Chen M.D.[1#], Hai Zhu M.D.[2#], Kai Zhang M.D., M.S.[1], Kangwu Chen M.D.[1*],

and Huilin Yang M.D., Ph.D.[1,3*]

[1]Department of Orthopaedics, The First Affiliated Hospital of Soochow University, No. 188 Shizi Street, Suzhou, Jiangsu215006, P.R. China

[2]Department of Orthopaedics, Affiliated Hospital of Nantong University, Xisi Road 20, Nantong, Jiangsu226001, P.R. China

[3]Institute of Orthopaedics, Soochow University, No. 708 Renmin Road, Suzhou, Jiangsu215006, P.R. China

[#]Hao Chen and Hai Zhu contributed equally to this study

***Corresponding authors:**

Huilin Yang

Email: suzhouspine@hotmail.com

Tel & fax: +86-0512-67780111

Kangwu Chen

Email: kangwuchen2008@hotmail.com

Tel & fax: +86-0512-67780111

Supplemental Figure 1:
ERα staining of LFJ

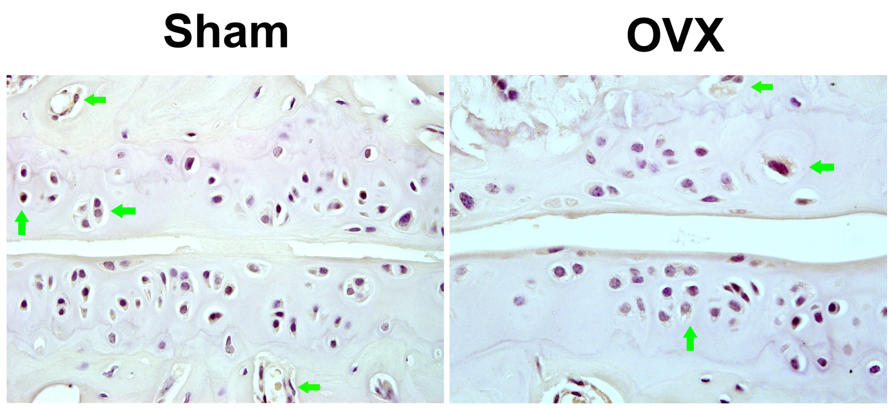

Green arrows pointed out some of the ERα positive cells in lumbar facet joint.

Supplemental Figure 2:
Grip force assays of different groups.

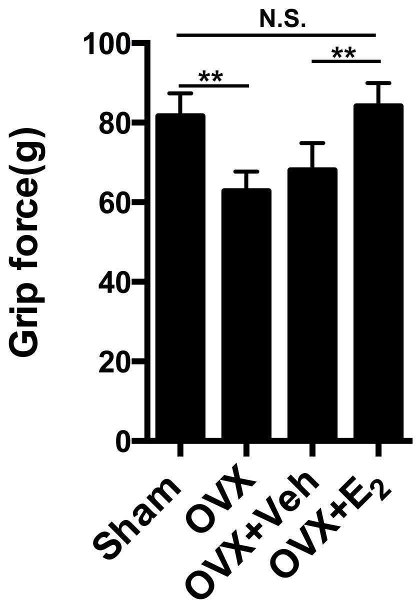

Supplement: Supplementary file 1 — Supplemental figures [file 41598_2017_1427_MOESM1_ESM.pdf]
